# Neuropsychological functioning and academic abilities in patients with juvenile idiopathic arthritis

**DOI:** 10.1186/s12969-021-00541-1

**Published:** 2021-04-14

**Authors:** Marine Granjon, Odile Rohmer, Nadège Doignon-Camus, Maria Popa-Roch, Christine Pietrement, Nathalie Gavens

**Affiliations:** 1 Department of Psychology, Laboratoire de Psychologie des Cognitions, University of Strasbourg, (LPC UR 4440), 12 Rue Goethe, FR-67000 Strasbourg, France; 2 Laboratoire Interuniversitaire des Sciences de l’Education et de la Communication (LISEC UR 2310), University of Strasbourg, Strasbourg, France; 3 Department of Pediatrics, CHU Reims, University of Reims Champagne-Ardenne, Reims, France; 4 Laboratoire Interuniversitaire des Sciences de l’Education et de la Communication (LISEC UR 2310), University of Haute-Alsace, Mulhouse, France

**Keywords:** Juvenile idiopathic arthritis, Central nervous system, Neuropsychology, Academic achievement

## Abstract

**Background:**

The involvement of the central nervous system is not rare in rheumatoid diseases. Even though children with juvenile idiopathic arthritis (JIA) may face academic difficulties until adulthood, very few studies have evaluated potential cognitive disorders in these patients. The present research aims to thoroughly investigate the cognitive and neuropsychological functioning of these patients.

**Methods:**

We measured the cognitive profile of JIA patients via their neuropsychological profile, implicit memory and social cognition skills, and estimated their academic performance using reading and mathematics tests. We recruited 21 children with JIA aged 6 to 17 years-old (*M* = 11.01, *SD* = 3.30) and 21 healthy children matched in age, gender, academic level (same school class) and socioeconomic status.

**Results:**

Our results showed that the cognitive profile and estimated academic ability of JIA patients are similar to those of their peers. These results support the hypothesis that children with JIA have the same cognitive predispositions to succeed at school as any other pupil.

**Conclusion:**

Comparing our results with the existing literature, we propose complementary hypotheses for further research. Longitudinal studies seem to be necessary to understand the psychosocial and cognitive processes involved in the development of children with JIA.

## Background

Juvenile idiopathic arthritis (JIA) is a chronic rheumatoid disease that occurs in children under 16 and affects approximately 32.6/100,000 children in Western populations [1]. JIA is a disease that significantly alters the quality of life and can lead to a disability that can persist into adulthood [2]. A good way to understand the impact of this illness on the patients’ daily life is to investigate to what extent these children are integrated in school. Very few studies have explored this topic in the literature.

The involvement of the central nervous system (CNS) is not rare in paediatric rheumatic diseases, including in JIA [3]. The existing literature on JIA suggests the possibility of CNS damage due to the inflammation itself, to long-term medication or to pain linked to the disease. Indeed, certain types of JIA may result in compression of the upper cervical cord or the brainstem closely related to the development of neurological deficits [4–6]. Beyond the risks caused by the inflammation itself, evidence has been put forward that neural impairments caused by the prolonged intake of medication - for example, corticosteroids, immunosuppressive drugs, or biotherapy are frequently prescribed as treatment independently or in combination [7–9]. Moreover, one of the recurring symptoms common to the different clinical manifestations of JIA is chronic pain [10]. Extended experience of pain may saturate cognitive resources, impair neuroplasticity and deregulate the activity of several chemical and cellular neuromediators [11]. Electroencephalographic studies put forward that patients with JIA have reduced sleep quality [12, 13], while quality sleep has been shown to be essential for the consolidation of information in the long-term memory [14, 15]. Finally, social adjustment and regulation of emotions appeared to be fragile in JIA patients [16, 17]. Nonetheless, to the best of our knowledge, there has been no research into the social cognition abilities of JIA patients, namely emotion recognition or theory of mind.

Since school achievement depends to a great extent on cognitive functions [18], the aim of the present study was to contribute to the literature on paediatric rheumatoid disease by investigating the neuropsychological profile of JIA patients, in order to assess their potential academic achievement. In this respect, it has been shown that inclusive education is both academically and socially more complicated for children with JIA, resulting in a higher rate of failure than among their peers [19]. While the drop in academic results was first attributed to psychosocial factors due to the burden of chronic illness [20], very few studies have examined the real abilities of these students to succeed at school. To fill this gap, we assessed whether the neuropsychological functioning and academic skills children with JIA differ from those of the general population. For a comprehensive assessment, we explored six domains: general intellectual efficiency (i.e., logical reasoning), attention and executive functions (i.e., cognitive adaptive capacity), language functions (i.e., ability to understand and produce language), implicit and explicit memory (i.e., ability to encode, store and retrieve information in memory, and this, when the instruction is stated explicitly or not), verbal and nonverbal learning (i.e., learning words or figures), visual-spatial treatments (i.e., speed and quality of visuospatial information processing) and social cognition (i.e., ability to put oneself in the others’ shoes). Mathematics and reading tests were used to estimate the academic capacities of the children with JIA.

## Methods

The study was pre-registered on Open Science Framework and all data are available on the following link: https://osf.io/g63ak/?view_only=d00cd687af2e430ca569074ae67445fe

### Participants

Twenty-one patients with JIA who met international classification criteria [21] were recruited. The participants with JIA were 16 girls and 5 boys, aged 6 to 17 (*M* = 11.01, *SD* = 3.30). Their performance in cognitive tasks was compared to that of 21 control participants with mean age of 10.9 years (*SD* = 3.11), who were the real classmates of the JIA patients, matched in gender, chronological age, socioeconomic status and academic level (Table 1). The socioeconomic status of the children was calculated according to the SEPI (Socio-Economic Position Index) [22], which accounts for the age, education level and professional category of the parent with the highest score. The mean disease duration of the patients was 4.82 years (*SD* = 2.69, range, 1.08 to 10.75 years). At the time of the experiment, 16 patients had an active disease, five were in remission, and all were on medication (i.e., antimetabolites and/or biological agents). The exclusion criteria were left-handedness and history of neurological deficits (e.g., stroke, head trauma).

**Table 1:** Demographic and clinical characteristics of the participants

	JIA (***N*** = 21)	Controls (***N*** = 21)	JIA vs. Controls
	Mean (standard deviation)	Mean (standard deviation)	
**Gender** (female: male ratio)	16: 5	16: 5	
**Age** (years)	11.01 (3.30)	10.90 (3.11)	*t*_*40*_ = 0.11, *p* > .05
**Education** (number of years after year 1)	4.29 (3.15)	4.29 (3.15)	
**Socioeconomic status** (SEPI; *N*)	74.00 (14.38)	71.24 (14.19)	*t*_*40*_ = 0.63, *p* > .05
Lower class	0	0	
Lower-middle class	2	4	
Middle class	3	5	
Upper-middle class	6	4	
Upper class	10	8	
**JIA subtype** ^a^ (*N*)			
Oligoarticular JIA	7		
Polyarticular JIA	8		
Enthesitis-related JIA	6		
Systemic JIA	0		
**Duration of the disease**^a^ (years)	4.82 (2.69)		
**Painful joints**^a^ (number)			
None	3		
1 joint	4		
2–4 joints	9		
> 4 joints	6		
**Medication** ^a^ (*N*)			
None	1		
Nonsteroidal anti-inflammatory drugs	6		
Disease-modifying anti-rheumatic drugs	10		
Biotherapies	17		
Corticosteroids	1		
**Diseases activity**^a^			
Self-reported, range 0–100	37.15 (range 0–83)		

^a^at evaluation time point

### Procedure and study design

Data were collected all over France. The experiment took place at the patient’s family home in a quiet room and lasted about 2 h. The children with JIA and the controls performed the experiment on the same day, one child in the morning and the second child in the afternoon, in a counterbalanced way. The two groups of participants used the identical battery of tests. The test involved a general assessment of the cognitive functions and an estimation of their academic achievement. The neuropsychological functioning was evaluated through a series of cognitive tasks divided into three domains: (1) the assessment of intellectual efficiency from the Raven’s Progressive Matrices [23], (2) the general battery of the NEPSY-II [24] was used to cover the main cognitive functions, and subtests of social cognition abilities; (3) an assessment of implicit memory (adapted verbal [25] and nonverbal [26] tasks). To complete the examination of the cognitive profile, academic performance was assessed through standardized tests: a reading task (Alouette’s test) [27] and a math task (Arithmetic subtest from WISC V) [28]. Potential interference between the different cognitive tasks was controlled by imposing a precise order of tasks.

### Experimental material

The tests used and their correspondence to the different cognitive processes are summarised in Table 2.

**Table 2:** Cognitive profile, academic estimated performance and number of participants with a deficit within each task for patients and controls

	JIA		Controls		JIA vs. Controls^*^
	Mean (standard deviation)	*Number of deficit participants*	Mean (standard deviation)	*Number of deficit participants*	
**Neuropsychological functioning**					
***General intellectual efficiency*** *(raw score)*					
Raven’s Progressive Matrices	37.62 (11.42)	*0*	37.48 (11.39)	*0*	*t*_*40*_ = 0.04, *p* > .05
***Main cognitive functions*** *(standard range 0–19)*					
Attention and executive functions					
Auditory Attention	9.10 (3.12)	*4*	9.72 (3.64)	*7*	*t*_*40*_ = −0.59, *p* > .05
Response Set	10.90 (3.95)	*0*	11.75 (3.13)	*0*	*t*_*40*_ = −0.76, *p* > .05
Word List Interference – Recall	7.55 (3.85)	*5*	8.35 (4.00)	*5*	*t*_*40*_ = −0.65, *p* > .05
Narrative Memory – Free recall	8.19 (3.12)	*8*	9.19 (3.88)	*7*	*t*_*40*_ = − 0.92, *p* > .05
Language functions					
Speeded Naming	10.67 (2.42)	*1*	10.00 (3.51)	*1*	*t*_*40*_ = 0.72, *p* > .05
Comprehension of Instructions	10.95 (1.99)	*0*	10.91 (2.77)	*2*	*t*_*40*_ = 0.06, *p* > .05
Memory and learning functions					
Narrative Memory – Cued recall	5.29 (2.10)	*19*	4.91 (2.34)	*17*	*t*_*40*_ = 0.55, *p* > .05
Memory for Faces	10.24 (2.76)	*4*	10.95 (2.16)	*0*	*t*_*40*_ = −0.94, *p* > .05
Memory for Faces Delayed	9.91 (3.53)	*7*	11.24 (2.97)	*1*	*t*_*40*_ = −1.32, *p* > .05
Visual-spatial treatments					
Geometric Puzzle	12.10 (2.93)	*1*	11.52 (3.31)	*3*	*t*_*40*_ = 0.59, *p* > .05
Social cognition					
Theory of Mind	8.76 (4.37)	*7*	10.05 (5.95)	*7*	*t*_*40*_ = −0.80, *p* > .05
Affects Recognition	9.43 (3.66)	*5*	9.38 (3.69)	*1*	*t*_*40*_ = 0.04, *p* > .05
***Implicit memory*** *(composite score)*					
Word Completion Test	3.24 (2.63)	*–*	3.48 (2.36)	*–*	*t*_*40*_ = − 0.31, *p* > .05
Fragmented Picture Test	10.14 (6.24)	*–*	10.91 (4.19)	*–*	*t*_*40*_ = − 0.41, *p* > .05
**Estimated academic performance**					
Reading skills *(z score)*					
Alouette-R	250.98 (147.20)	*1*	248.84 (118.83)	*2*	*t*_*40*_ = 0.05, *p* > .05
Mathematical skills *(standard range 0–19)*					
Arithmetic	9.52 (2.77)	*4*	9.86 (2.61)	*3*	*t*_*40*_ = −0.40, *p* > .05

^*^Statistical analysis was performed using a *Student’s t-test* for paired sample, allowing to compare the means of two matched sample groups. If the *p*-value is above .05, the mean difference between the two groups is not statistically significant

#### Neuropsychological functioning

##### General intellectual efficiency

Raven’s Progressive Matrices [23] were selected to assess nonverbal reasoning. This test has been validated among people of all ages all over the world. It is for the participant to complete a series of figures by choosing among several the corresponding item. This task requires at each stage cognitive skills to analyse information with progressive difficulty. The number of correct responses is counted at the end of the test. The raw scores are then compared to the norm scores corresponding to each participant age groups, and transformed into percentiles. Deficit score are considered below percentile 10 (i.e., cut-off). This means that participants who obtained these results have significantly lower general intelligence performance than the general population.

##### Main cognitive functions

Eleven subtests of the French version of the NEPSY-II24 were used, standardized for children and adolescents aged from 6 to 17. (1) Auditory Attention assesses auditory selective and sustained attention. (2) Response Set follows the previous task and involves new rules that require shifting and inhibition skills. (3) Word List Interference measures short-term memory and working verbal memory facets. (4) Narrative Memory assesses both organisational strategy skills of executive functions over the free recall condition and long-term verbal memory over the cued recall condition. (5) Memory for Faces evaluates short-term visual memory via the encoding of facial features as well as face discrimination and recognition. (6) Memory for Faces Delayed repeats the same procedure 15 to 25 min later to assess long-term storage of visual memories. (7) Speeded Naming measures phonologic and semantic access abilities (i.e., units that make up the word and its meaning), including stimuli such as the names of colours, shapes, sizes, letters or numbers based. (8) Comprehension of Instructions evaluates language skills and the ability to perceive process. (9) Geometric Puzzle involves processes of mental rotation, visual-spatial analysis, and global/local perception. (10) Theory of Mind assesses the ability to understand mental states and the perspective of another person. (11) Affects Recognition measures the ability to recognize emotions (i.e., neutral, joy, anger, disgust, sadness or fear).

The different scores obtained in the NEPSY-II subtests were transformed into scaled scores (*M* = 10, *SD* = 3; range, 1 to 19) with respect to the normative values for the corresponding chronological age [24]. A score below 7 indicates a deficit (i.e., cut-off).

##### Implicit memory

**Fig. 1:**
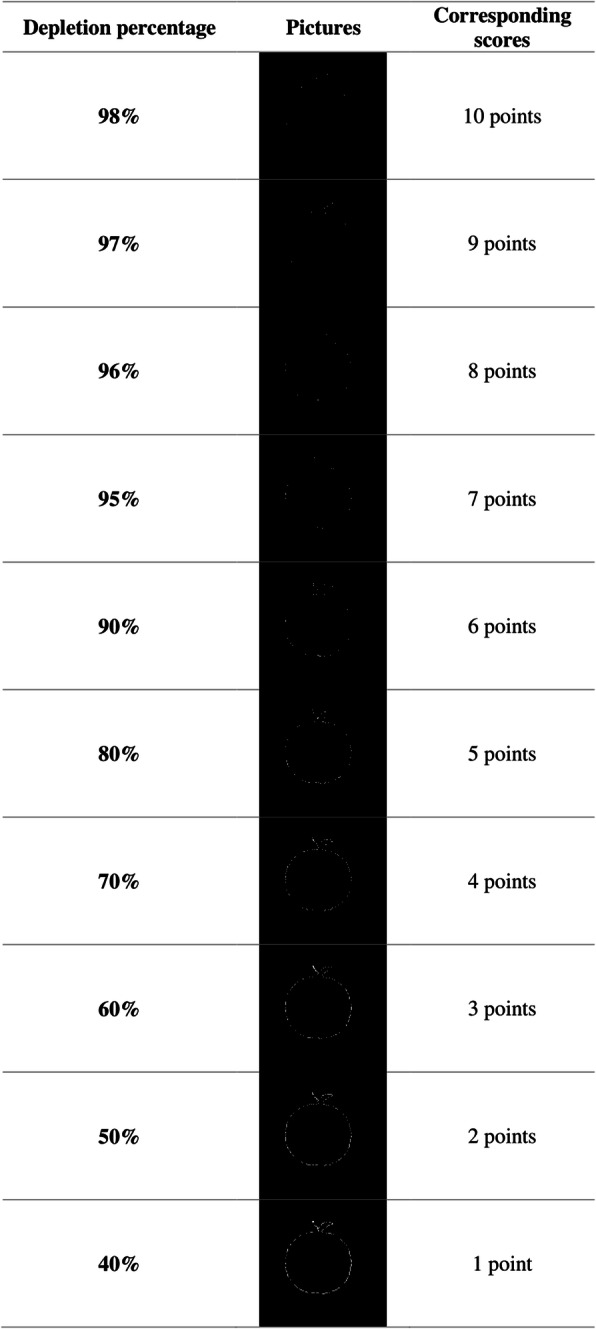
Example of the “Apple” picture, depletion level and corresponding recognition score

In addition to the standardized tests, the ability to implicitly encode, store and retrieve information in memory was tested through a word completion task and a fragmented picture task. The two tasks were adapted for native French-speaking children aged from 6 to 17. Word Completion Test (adapted from a previous research) [25] investigates implicit verbal memory by measuring the participants’ capacity to recall words that were previously encoded involuntarily, among primed (List A) versus non-primed words (List B). Fragmented Picture Test (adapted from a previous research) [26] assesses implicit nonverbal memory like in the previous task. In this study, the material was borrowed from a study [29] employing the pictures in the original Gollin test [26] modified by a Gabor filter, which make it possible to obtain pictures of a high level of biological plausibility. The stimuli are from the most fragmented version to the least fragmented version, according to 10 levels of depletion (Fig. 1) [29]. The reported scores indicated the stage of fragmentation corresponding to the time taken to recognize the picture as belonging to list A and B. A pre-test was performed before the study to check the validity of the tasks. To analyse implicit memory capacities, a hybrid score was obtained through the following calculation for both tasks: list A (primed stimuli) - list B (non-primed stimuli). Primed items are expected to be recognized faster than non-primed items, both for the JIA participants and their peers.

#### Estimated academic performance

Participants performed two reading and mathematics standardized tasks, commonly used to estimate the academic performances [30]. Alouette-R [27] is a standardized test requiring the subject to read a meaningless text of 265 words aloud within a 180 s time window. The reading efficiency measure (calculated by multiplying the number of accurately pronounced words by 180 then dividing this by the time it took the subject to read the text in seconds) indicates the speed-accuracy trade-off in word processing. Then, this score is compared to the general population and transformed into a *z* score (i.e., number of standard deviations from the mean value of the reference population, range − 3 to 3). The deficit cut-off is considered to be − 1.65. Arithmetic (WISC-V subtest) [28] assesses academic math skills. The raw data were transformed into scaled scores according to the standards of the WISC-V (rage, 1 to 19) [28]. Similar to the NEPSY, the deficit cut-off score is 7. Obtaining an estimation of academic performances using standardised tasks has the advantage of overcoming the bias inherent to the school context (e.g., teacher’s perception, class effect) [31].

## Results

The two groups did not differ in terms of age, gender, socioeconomic background (Table 1), and there was no impact of these variables on the measures (all *p* > .05).

### Neuropsychological functioning

No statistical difference was found between the two groups in any of the measurements of cognitive processes (Table 2). Note that all scaled tasks were found to be within the standard range, with the exception of Narrative Memory (cued recall condition, *M*_*JIA*_ = 5.29, *SD*_*JIA*_ = 2.10; *M*_*controls*_ = 4.91, *SD*_*controls*_ = 2.34), which was below the average score (i.e., *M* = 9, *SD* = 2), in the same way in the two groups. The results also showed that both groups presented preserved implicit memory abilities recognisable by stronger activation of primed than non-primed stimuli in verbal modality (JIA, *t*_*40*_ = 5.65, *p* < .001; Controls, *t*_*40*_ = 6.75, *p* < .001) and nonverbal modality (JIA, *t*_*40*_ = 7.45, *p* < .001; Controls, *t*_*40*_ = 11.91, *p* < .001). Regarding the number of participants performing significantly low to each task, the distribution appears roughly equivalent, with the exception of Memory for Faces, Memory for Faces Delayed and Affects Recognition. Participants with JIA appear more numerous than their peers to have low scores of long-term memory and recognition of emotions. A non-parametric statistical analysis using Kruskal-Wallis’s test indicated that these difficulties were evenly distributed among the JIA subtypes (i.e., oligoarticular, polyarticular and enthesitis-related JIA, Table 3).

**Table 3:** Cognitive profile and academic estimated performance depending on JIA subtypes

	Oligoarticular JIA	Polyarticular JIA	Enthesitis-related JIA	Deficit cut-off score	Oligoarticular vs. Polyarticular vs. Enthesitis-related JIA^*^
	Mean (range)	Mean (range)	Mean (range)		
**Neuropsychological functioning**					
***General intellectual efficiency*** *(raw score)*					
Raven’s Progressive Matrices	37.86 (23–47)	32.13 (14–57)	32.13 (14–57)	P10	X^2^ = 4.83, *p* > .05
***Main cognitive functions*** *(standard range 0–19)*					
Attention and executive functions					
Auditory Attention	11.00 (8–14)	8.57 (7–12)	9.00 (5–12)	7	X^2^ = 2.95, *p* > .05
Response Set	11.60 (11–13)	12.57 (12–14)	12.00 (9–14)	7	X^2^ = 1.24, *p* > .05
Word List Interference – Recall	8.00 (5–10)	9.86 (7–14)	7.00 (1–12)	7	X^2^ = 3.74, *p* > .05
Narrative Memory – Free recall	7.00 (4–11)	8.00 (2–12)	9.83 (6–16)	7	X^2^ = 3.20, *p* > .05
Language functions					
Speeded Naming	10.57 (7–14)	10.25 (8–14)	11.33 (8–15)	7	X^2^ = 0.77, *p* > .05
Comprehension of Instructions	10.86 (9–15)	10.75 (9–13)	11.33 (9–14)	7	X^2^ = 2.95, *p* > .05
Memory and learning functions					
Narrative Memory – Cued recall	5.86 (3–10)	5.50 (3–9)	4.33 (3–7)	7	X^2^ = 1.98, *p* > .05
Memory for Faces	10.14 (7–14)	10.00 (6–15)	10.67 6–14)	7	X^2^ = 0.92, *p* > .05
Memory for Faces Delayed	9.29 (4–15)	10.75 (6–15)	9.50 (5–15)	7	X^2^ = 2.77, *p* > .05
Visual-spatial treatments					
Geometric Puzzle	14.00 (9–18)	10.38 (7–15)	12.17 (9–14)	7	X^2^ = 4.92, *p* > .05
Social cognition					
Theory of Mind	9.43 (4–16)	7.38 (1–11)	9.83 (1–16)	7	X^2^ = 1.11, *p* > .05
Affects Recognition	10.71 (7–14)	7.50 (1–14)	10.50 (7–13)	7	X^2^ = 2.22, *p* > .05
***Implicit memory*** *(composite score)*					
Word Completion Test	3.29 (−1–7)	2.88 (− 1–6)	3.67 (2–5)	–	X^2^ = 0.21, *p* > .05
Fragmented Picture Test	7.57 (0–12)	13.38 (2–29)	8.83 (2–15)	–	X^2^ = 4.54, *p* > .05
**Estimated academic performance**					
Reading skills *(z score)*					
Alouette-R	−0.35 (− 1.7–0.86)	− 0.40 (− 1.17–0.68)	0.10 (− 1.70–0.96)	- 1, 65	X^2^ = 1.68, *p* > .05
Mathematical skills *(standard range 0–19)*					
Arithmetic	11.14 (8–14)	9.88 (4–13)	7.17 (3–10)	7	X^2^ = 8.31, *p* < .05

^*^Statistical analysis was performed using a non-parametric test, *Kruskal-Walllis*, due to the small sample size of the JIA groups. If the *p*-value is below .05, the mean difference between the two groups is statistically significant

### Estimated academic level

No significant difference was observed between children with JIA and controls in reading (Alouette-R task) and mathematics (Arithmetic task). This result is confirmed by the number of participants who performed below the cut-off score in the two groups (Table 2). Comparisons between JIA subtypes indicated a significant difference in Arithmetic, explained by the fact that participants with an oligoarticular JIA (*M* = 11.14) performed better than participants with enthesitis-related JIA (*M* = 7.17, Table 3).

## Discussion

The aim of the present study is to contribute to the literature on childhood rheumatoid disease by thoroughly investigating the cognitive profile of the children with JIA and estimating the potential for academic achievement of JIA patients. Our sample comprised only non-systemic forms with a dominance of polyarticular and oligoarticular JIA forms, which may explain the over-representation of girls [32]. It is important to note that to the best of our knowledge, this is the first research to investigate the cognitive functions in non-systemic JIA patients. Our results highlighted preserved cognitive abilities, social cognition and implicit memory, and similar skills required to succeed academically in JIA patients and their peers. Importantly, this is the first time social cognition and implicit memory have been assessed in this population of patients. Indeed, an early social cognition, defined as the ability to understand other people’s points of view and feelings, predicts children’s subsequent school achievement and this pathway is mediatised by children’s level of social competence [33]. Moreover, implicit memory is also essential in learning and robust to cognitive disturbances. Research has shown preserved implicit memory in elderly and children with mental retardation [34, 35]. As the population of JIA children could have cognitive impairments, exploring implicit memory is essential. In order to thoroughly measure verbal and nonverbal implicit memory, we used experimental priming paradigms leading us to create two new tasks, a word completion task and a fragmented picture tests. The results of the cognitive profile and estimated academic achievement appear to be consistent since the cognitive functions are an important predictor of academic achievement, especially general intelligence and working memory [18]. Our results stand in apparent contradiction with literature that suggests impaired life conditions in adult patients with JIA. Three complementary interpretations may account for this discrepancy.

First, the developmental perspective according to which cognitive impairments develop later in a patient’s life should be taken into consideration. Our results are in line with a previous study indicating a normal intellectual quotient in children with systemic JIA [20], even though another body of evidence suggests potential neurocognitive impairment in these patients, whether directly or indirectly linked to the clinical manifestation of the disease process [3]. Specifically, the maturation of the CNS is subject to the consequences of various symptoms of JIA including the inflammation inherent in JIA [4–6], the long-term medication [7–9], reduced quality of sleep [12, 13], as well as the pain related to the disease [11]. In 2014, a review put forward the idea that children with JIA experience long-term changes to the CNS that could last into adulthood, resulting in lowering of the threshold of pain tolerance often associated with the inflammation [36]. In this perspective, a research assessed the cognitive aptitudes of 121 adult patients with rheumatoid arthritis [37]. Their results indicated that chronic pain, mediatised by depression, leads to impairment of cognitive functions, particularly the speed of information processing, reasoning ability, working memory and long-term memory. Importantly, these consequences depend on the duration and severity of the disease [38]. This set of findings is consistent with our results in assuming that while children with JIA are cognitively preserved, neuropsychological impairment may manifest itself later due to a lasting alteration of the CNS.

This hypothesis is supported by our descriptive data suggesting that some cognitive weaknesses begin already to appear in the JIA participants. Indeed, although the means comparison does not indicate a significant difference between the two groups, the number of participants having a deficit is higher in tasks requiring memory capacities and emotion recognitions. Yet, these two processes are essential for learning and academic success [15, 33]. If these descriptive results should be taken with caution at this point, they are nonetheless interesting. Indeed, the small size of our sample induces little statistical power and there is a risk of not detecting a difference between the two groups while existing in reality (i.e., statistical type 2 error). Furthermore, the comparison between JIA subgroups revealed that the enthesitis-related JIA group is more likely to underperform in mathematics compared to the oligoarticular JIA group. Future research should scrutinize in more depth the differences in prognosis for academic achievement as a function of the JIA subtypes.

Second, in addition to the neurological factors, the psychosocial determinants should be considered from a developmental perspective. Our results on the estimation of school achievement are only based on standardized literacy and mathematic tasks, which might not reveal the real school achievement of such children. It has been shown that, at most, 50% of the variance in academic outcomes was explained by the cognitive abilities of the students [18]. In other words, psychosocial factors may affect the deployment of the full cognitive capacities of JIA patients in a non-negligible way. Two factors are identified in the literature as particularly important. In the first place, the psychological resources required to cope with a chronic illness were investigated in JIA patients mostly based on the parents’ reports [16, 17]. The results mainly put forward that the burden of a chronic disease has a negative impact on emotional adjustment to social life [39], including school [20, 40]. Moreover, the constraints of coping with the disease and its symptoms means these children are more often absent from school than their classmates, disrupting both learning and their integration in the class Consequently, students with JIA are twice as likely to fail at school than their peers [19]. Next, a substantial literature on social psychology has highlighted subtle negative reactions from teachers towards the inclusion of students with disability [41, 42]. Yet the teacher’s perceptions have an important impact on students’ academic success [31], especially when the disability is invisible [43] as is the case with JIA. While academic achievement is an important predictor of well-being in adulthood [44], the quality of life of adults with longstanding JIA was found to hampered and was accompanied by a higher unemployment rate than in the general population [38]. All these studies support the hypothesis that JIA children face subtle psychosocial barriers to the full expression of their cognitive abilities in the long term. As our sample is small and did not allow us to test the above-mentioned developmental hypotheses, we strongly encourage further research to deepen the two developmental perspectives put forward. Research is also needed to more directly explore the factors that contribute to the emergence of elements that may threaten the quality of life (e.g., age, socio-economic status, perception of disability). Longitudinal methodologies would be particularly suitable since they would enable investigation of the precise factors leading to the deterioration.

Third, the clinical and socio-demographic characteristics of the patients with JIA may shed light on the results obtained in this research. Indeed, systemic forms of JIA are absent from our sample of participants. Yet, they are the most severe and most likely to affect the CNS due to the inflammation or the macrophage activations syndrome [45]. Thus, our results can only be relevant for non-systemic JIA. Future research should specifically focus on exploring the cognitive profile of systemic JIA, since it appears to be different in prognosis compared to other forms of JIA. Moreover, the majority of participants were on disease-modifying anti-rheumatic drugs and biotherapy at the time of the evaluation. If the latter might impact the SNC on the long term [9], it does not involve an important risk which might be the case for the corticosteroids. Indeed the corticosteroids were shown to disrupt the hippocampus and frontal lobe - brain regions responsible for memory, learning, as well as involved in cognitive and behavioural control [46, 47]. The low intake of corticosteroids in our sample may be related to the absence of systemic JIA. Finally, it should be noted that the patients were recruited on voluntary basis, mainly through the association of patients to which they are affiliated. Thus, the participants’ family motivation to participate in our research may have induced a recruitment bias. The sociodemographic data confirm that most of the children with JIA in our study came from upper-middle and upper class families. For this reason, it is difficult to extrapolate our results to all children with JIA, since our sample was small and the majority of the patients were recruited on a voluntary basis. While this did not bias our results as the JIA and the control groups were matched, it may have skewed the results by representing predominantly socially advantaged backgrounds contributing to better academic outcomes. Indeed, the family’s socioeconomic status is known to play an important role in a child’s academic achievement [48], and may explain why JIA children performed as well as their control peers in the academic tasks. Thus, following our first two interpretations, our results suggest that these children are less at risk of developing cognitive disorders later if they have such strong support from their families than most patients with JIA.

## Conclusion

The results of this study revealed no significant differences between the JIA group and the control group, either the complete neuropsychological assessment or the tasks estimating academic performance. The two groups we compared were perfectly matched in age, gender, academic level (same-grade classrooms) and socioeconomic status. While our research indicates that children with JIA have every chance of developing positively, several studies have pointed out that, as adults, they are more prone to reduced quality of life [37], or even to developing cognitive impairments [36]. One of the interpretation put forward in this article is that medical (e.g., treatment), psychological (e.g., mental state related to the chronic pain), and sociocognitive (e.g., attitudes toward disability) factors may progressively crystallize long-term difficulties likely to weaken the quality of life of these patients. Our results emphasize the importance of supporting JIA patients and accompanying them throughout their education, which appears to be crucial for their future quality of life. Indeed, a deteriorated psychological state has been shown to have a negative impact on the cognitive functioning of people with JIA [36]. Further work is needed to better understand the development of the processes involved in the discrepancy between cognitive abilities in children and in adults with rheumatoid arthritis disease.

## Data Availability

The study was pre-registered on Open Science Framework and all data are available on the following link: https://osf.io/g63ak/?view_only=d00cd687af2e430ca569074ae67445fe.

## Abbreviations

JIA: Juvenile idiopathic arthritis
CNS: Central nervous system
